# Rheumatic Manifestations of Sarcoidosis

**DOI:** 10.3390/diagnostics14242842

**Published:** 2024-12-17

**Authors:** Julia Day, Philip D. H. Hamann

**Affiliations:** 1 North Bristol NHS Foundation Trust, Bristol BS10 5NB, UK; philip.hamann@nbt.nhs.uk; 2 Musculoskeletal Research Unit, University of Bristol, Southmead Hospital, Learning and Research Building, Southmead Road, Bristol BS10 5NB, UK

**Keywords:** sarcoidosis, arthritis, myopathy, Lofgren’s, rheumatology

## Abstract

Sarcoidosis is a multisystem granulomatous inflammatory disorder, of unknown aetiology, which causes a wide spectrum of clinical phenotypes. It can present at any age, most commonly between 20 and 60 years, with a roughly equal sex distribution. Diagnosis is often delayed due to multiple diagnostic mimics, particularly joint disease. Common presenting features include pulmonary disease, with bilateral hilar lymphadenopathy and pulmonary infiltrates, cutaneous lesions, and ocular disease. Musculoskeletal manifestations are reported in 10–40% of patients with sarcoidosis and include bone lesions, acute arthritis, chronic arthritis, axial disease, dactylitis, and sarcoid myopathy, which are explored in detail in this review article. Diagnosis is confirmed through histological evidence of non-caseating granuloma on tissue biopsy. Newer imaging modalities, including ^18F^FDG PET/CT, can help identify the extent of musculoskeletal involvement, and biomarkers can provide weight to a diagnosis, but there is no single biomarker with prognostic value for disease monitoring. The mainstay of treatment remains corticosteroids, followed by disease-modifying antirheumatic drugs such as methotrexate and antimalarials. More recently, biologic treatments have been used successfully in the treatment of sarcoidosis with rheumatic involvement.

## 1. Introduction

Sarcoidosis, first described in the late 19th century, is a multisystem granulomatous inflammatory disorder which can present with a wide spectrum of clinical phenotypes. The pathognomic hallmark is the infiltration of various organs by non-caseating granulomas. Despite the improved understanding of the pathogenesis of sarcoidosis, the underlying cause remains unclear [1]. Musculoskeletal (MSK) manifestations are seen in up to a third of patients with sarcoidosis, and include joint, bone, and muscle involvement. The clinical spectrum of disease is broad, ranging from mild arthralgia to widespread destructive bone and joint disease. Joint involvement tends to be part of the initial presentation of sarcoidosis, occurring early in the disease course. The aim of this review article is to provide an overview of the manifestations of sarcoidosis that are likely to present to, and be managed by, a rheumatologist.

### Epidemiology

The epidemiology of sarcoidosis is difficult to define, as many cases remain asymptomatic and epidemiologic assessment is hampered by the lack of a consistent case definition, a lack of diagnostic sensitivity, variations in diagnostic methods, access of patients to health care, and the geographic distribution of sub-phenotypes. Sarcoidosis occurs throughout the world, in every age group and ethnicity, but is noted to have higher incidence in African American and Scandinavian populations. The condition affects both sexes, with slight predominance in women. The incidence is reported to peak at about 30–50 years of age in men and 50–60 years of age in women [2,3]. Global incidence has been estimated to be 1.0–35.5 per 100,000 individuals per year, and prevalence between 4.7 and 64 per 100,000—the wide variation in reporting may be due to the different ethnicities of populations studied and the variety of older and newer studies, as the disease becomes more recognised [1]. Joint involvement has been reported in 2 to 65% of patients with sarcoidosis, more commonly in women, and racial variation has been noted in studies Yeung et al., 2024 [4]. In around two-thirds of patients, the disease course is self-remitting over around 12–36 months. Up to one-third require longer-term treatment, with systemic treatment usually reserved for those with internal organ involvement or severe disease [2]. Patients with sarcoidosis have an increased mortality rate, often due to respiratory or cardiac involvement, with a shorter life expectancy than the general population [5]. 

## 2. Pathogenesis

**Figure 1:**
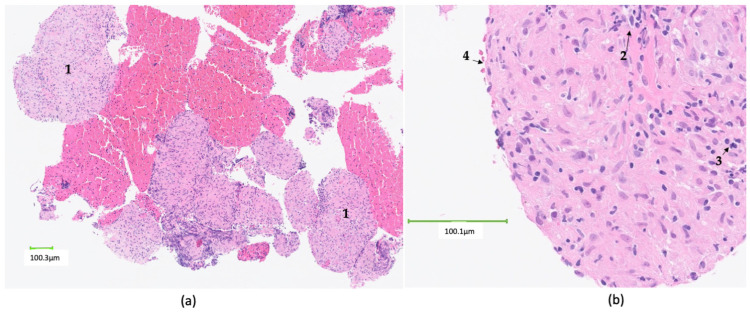
Microscopic appearances of non-necrotising granuloma from a biopsy of a hilar lymph node in a patient with confirmed systemic sarcoidosis. The patient presented with acute Lofgren’s syndrome with erythema nodosum, bilateral ankle swelling, and evidence of bilateral hilar lymphadenopathy on chest radiograph. (**a**) Microscopic appearance of non-necrotising granulomata (1), obtained from a hilar lymph node via endobronchial ultrasound-guided transbronchial biopsy; (**b**) Sarcoid granuloma demonstrating clustered epitheloid histiocytes (2) and multinucleated giant cells (3) surrounded by a rim of fibrosis (4).

The underlying aetiology remains unclear, but it is proposed that a genetic susceptibility and environmental factors contribute to disease development. In those with an immunogenetic predisposition, as-yet-unidentified environmental antigens appear to trigger a dysregulated immune response causing Th1 cytokine activation and infiltration of tissues with CD4+ lymphocytes and macrophages. Macrophages differentiate to form epitheloid cells and fuse into giant cells. Sarcoid granulomas are usually non-necrotising multinucleated giant cells, composed of clustered epitheloid histiocytes, and become surrounded by an outer rim of fibrosis, with surrounding CD8 T cells, mast cells, dendritic cells, and fibroblasts, as illustrated in Figure 1 [6,7].

## 3. Diagnosis

### 3.1. Diagnostic Mimics

Due to its broad range of possible presenting features, the diagnosis of sarcoidosis can often be challenging and, consequently, treatment may be delayed. A careful history and clinical examination are essential. Ultimately, the diagnosis is based on three major criteria: compatible clinical characteristics, identification of non-necrotising granulomas in one or more tissue samples, and the exclusion of other causes of granulomatous disease [8]. Other granulomatous diseases can mimic sarcoidosis both histologically and clinically, including infections (such as mycobacterial infection), granulomatous reactions to occupational and environmental exposures, drug reactions, lymphoproliferative disorders, systemic vasculitides, and idiopathic granulomatous conditions. As there are no standardised diagnostic criteria for sarcoidosis, it is possible that some of these sarcoidosis mimics may represent varied clinical presentations of sarcoidosis itself [9]. General systemic symptoms are common, including fatigue (in up to 70%), fever, and peripheral lymphadenopathy [10].

Joint disease in sarcoidosis can resemble reactive arthritis. In those presenting with bilateral ankle arthritis, it is important to look for additional evidence, such as bilateral hilar lymphadenopathy on chest radiograph and skin rashes, to avoid missing a diagnosis of sarcoidosis. Other possible causes of a new inflammatory mono/oligoarthritis involving the ankle include spondyloarthritis (more commonly than in rheumatoid arthritis) and gout. The ankle joint is often involved in septic arthritis, especially bacterial, chikungunya, and HIV infection, and much less commonly in tuberculosis. Primary osteoarthritis of the ankle is rare, and usually secondary to trauma. The ankle swelling seen in Lofgren’s syndrome is usually due to subcutaneous oedema rather than true synovitis [11]. Erythema nodosum, often presenting with joint symptoms, may also be seen in association with several other conditions including infections, medications, and other underlying diseases [12].

### 3.2. Investigation

Since the presenting features of sarcoidosis are often so widely variable and non-specific, histological confirmation is usually needed to secure the diagnosis. Investigation is targeted towards linking clinical and radiographic findings to histological evidence of non-caseating granuloma in organ tissue. No single diagnostic biomarker exists. Calcium in both serum and urine and angiotensin-converting enzyme (ACE) in serum are well-established clinical tools. Serum angiotensin-converting enzyme can be used for monitoring disease activity in the individual patient; however, it is only elevated in 41–58% of patients, and its diagnostic value is low due to its lack of sensitivity and specificity. A study which included a large proportion of patients with sarcoid arthritis also demonstrated an elevated serum ACE in around half of patients, but found that this did not correlate with disease severity [13]. Abnormal calcium metabolism in sarcoidosis patients can lead to hypercalcemia, hypercalciuria, and nephrocalcinosis. Hypercalcemia in sarcoidosis is seen in around half of patients, and is usually due to an increased activity of 1α-hydroxylase in macrophages of pulmonary granulomata, resulting in low levels of 25-hydroxyvitamin D and high levels of calcitriol [14].

Extrapulmonary manifestations are most often discovered during the diagnostic work-up of a pulmonary sarcoidosis. A subset of patients present with only extrapulmonary manifestations of sarcoidosis, and in this instance, fludeoxyglucose F18 positron emission tomography/computed tomography (^18F^FDG-PET/CT) may demonstrate an uptake in hilar or mediastinal lymphadenopathy, with subsequent endobronchial ultrasound and biopsy revealing granulomas [15]. ^18F^FDG-PET/CT changes are not specific for sarcoidosis, but it is sensitive when identifying bone and joint involvement in sarcoidosis, and useful for identifying potential targets for biopsy [16].

### 3.3. Potential Novel Diagnostic Tools

Whilst certain disease phenotypes portend a good prognosis, a large proportion of patients with systemic sarcoidosis would benefit from advances in biomarker development, both at diagnosis and for monitoring disease activity and informing prognosis. Recent advances in genome-wide association studies offer the potential to identify novel biomarkers which may be useful in diagnosis and prognostication (Casanova et al., 2015 [17]). Examples include chitotriosidase, originating from monocytes and macrophages, which has been associated with disease progression, and sIL-2R, which is lymphocyte-associated and associated with disease severity and extra-pulmonary organ involvement. More recently, a novel immunoassay for sarcoidosis was created, following the discovery of two novel immunoepitopes. With further development, this may prove to be a useful tool in helping to differentiate the MSK manifestations of sarcoidosis from other inflammatory rheumatic disorders (Peng et al., 2024 [18]).

The expansion of the use of ^18F^FDG-PET/CT in sarcoidosis is a highly valuable tool in staging and for the identification of occult sites and potential biopsy sites, although its use is limited by the cost and risk of radiation exposure. In the future, it may be more widely used to assess inflammatory active sarcoidosis in patients with prolonged symptoms, especially when other markers of the disease are within normal values. Future prospective multicentre studies are needed to refine the clinical applications of ^18F^FDG-PET/CT in patients with sarcoidosis (Sobic-Saranovic et al., 2013 [19]).

## 4. Rheumatic Manifestations

**Figure 2:**
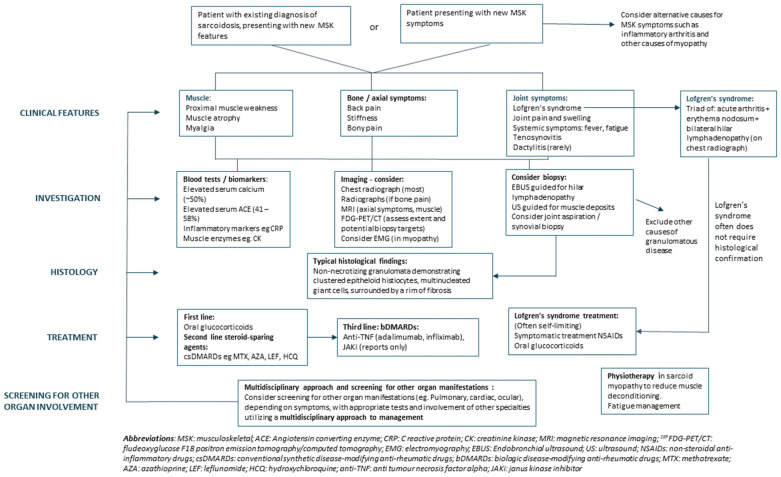
Flowchart of a practical approach for investigation and management of the MSK manifestations of sarcoidosis.

Non-specific, systemic symptoms are common in sarcoidosis and include arthralgia and fatigue [1]. In terms of joint disease, acute arthritis is the most common rheumatic presentation. This includes Lofgren’s syndrome—the triad of acute arthritis, erythema nodosum, and bilateral hilar lymphadenopathy. Less frequently, a chronic arthritis pattern is seen. Bony lesions and asymptomatic X-ray changes are common and muscle involvement with sarcoid myopathy is also well reported. Figure 2 provides an overview of a practical approach for the investigation and management of the MSK manifestations of sarcoidosis.

### 4.1. Acute Arthritis Including Lofgren’s Syndrome

**Figure 3:**
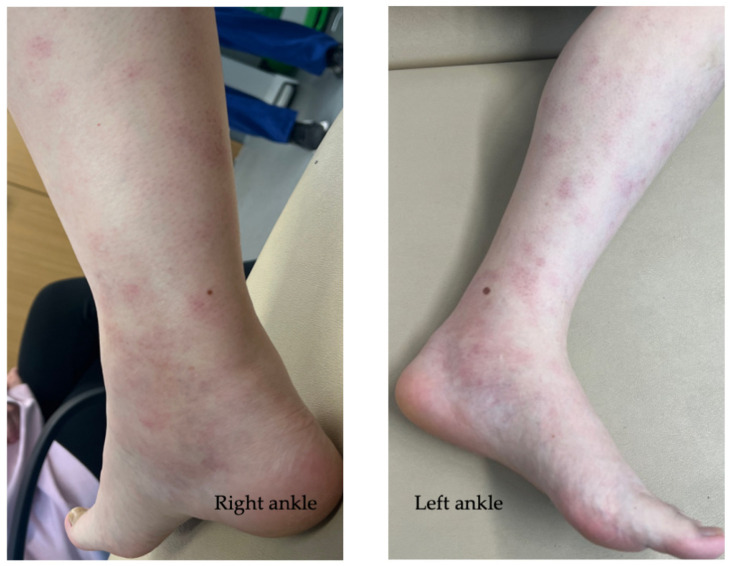
Ankle swelling and erythema nodosum in a patient with acute Lofgren’s syndrome (images included with patient consent).

Acute arthritis is the most common rheumatic manifestation of sarcoidosis, usually seen as part of its initial presentation [20]. It is predominantly a symmetrical, oligoarticular arthritis affecting the lower limbs, often presenting with bilateral ankle swelling [4]. Fever is a common finding in around half of those with acute sarcoid arthritis. Erythema nodosum is reported in around 40%, and found more commonly in Caucasians and women [21]. Asymptomatic bilateral hilar lymphadenopathy is found in 90% patients. Lofgren’s syndrome, first described by Swedish Professor Sven Lofgren in 1952, describes the triad of arthritis, erythema nodosum, and bilateral hilar lymphadenopathy seen on chest radiograph [22]. This combination of presenting features is so highly sensitive and specific for a diagnosis of sarcoidosis that it does not usually require histological confirmation [23]. The grade of radiographic bilateral hilar lymphadenopathy is usually lower in acute Lofgren’s syndrome than other forms of systemic sarcoidosis, indicating milder lung involvement [22]. Figure 3 illustrates ankle swelling and erythema nodosum in acute Lofgren’s syndrome.

Studies using ultrasound, including power Doppler studies, and magnetic resonance imaging (MRI) have consistently shown that much of the ankle swelling in acute sarcoid arthritis represents periarticular soft tissue inflammation, accompanied by small amounts of joint and tenosynovial fluid, without true synovitis nor evidence of hyperperfusion. This is often accompanied by tenosynovitis [24,25]. If histological confirmation is sought, synovial fluid analysis may show a mild inflammatory infiltrate with predominance of mononuclear cells, but may not demonstrate a granulomatous reaction in Lofgren’s syndrome [12].

Acute sarcoid arthritis tends to be self-limiting, with symptoms settling over the course of a few months, and it responds very well to treatment with non-steroidal anti-inflammatory drugs [21,26]. Lofgren’s syndrome has an excellent long-term prognosis, and joint destruction is very rarely seen. Relapse is infrequent, but more likely if glucocorticoids were required to induce remission in the first place [27].

Epidemiological studies have demonstrated a negative association between smoking and sarcoid arthritis. There appears to be a seasonal distribution, with clustering of presentations in the springtime [28]. It is proposed that there may be an immunogenetic predisposition to sarcoidosis, in particular sarcoid arthritis, as Human Leucocyte Antigens (HLA) appear to strongly influence the disease course. Strongly significant associations with acute sarcoid arthritis have been found for DQ2 (DQB1*0201) and DR3 (DRB1*0301), and confer an excellent prognosis with a high rate of spontaneous remission [21,29]. 

### 4.2. Chronic Sarcoid Arthritis

Chronic arthritis in sarcoidosis with a disease duration longer than 6 months is a rarer presentation (0.2–1.3% of sarcoidosis cases) [30,31]. It usually occurs in the context of more widespread systemic involvement, including parenchymal lung disease, skin disease, eye involvement, and tenosynovitis [32]. In one study, chronic joint involvement was found to be more prevalent in non-Lofgren’s syndrome, in those where specific treatment was required, in older age, and in those with more advanced chest radiograph findings [22]. Chronic sarcoid arthritis mostly presents as a persistent oligoarthritis or polyarthritis, most frequently affecting the hand joints, followed by the wrist, ankle, and knee joints, and more rarely involves the shoulder, hip, and elbow joints [20,33]. The differential diagnosis includes other causes of an inflammatory oligo- and polyarthritis such as rheumatoid and reactive arthritis. Sarcoid monoarthritis is rare, and should prompt the exclusion of other causes such as septic arthritis, gout, and calcium pyrophosphate deposition disease. Acute gout may also coexist in sarcoidosis, the diagnosis confirmed by the presence of monosodium urate crystals in synovial fluid [34].

Imaging findings in chronic sarcoid arthritis are non-specific. Radiographs may appear normal, but destructive forms can demonstrate joint space narrowing, the demineralisation of subchondral bone, and associated soft tissue infiltration. MRI may be considered in the evaluation of patients with sarcoidosis with joint symptoms if standard radiographs are negative, and often depicts tenosynovitis [35]. An examination of synovial biopsy in chronic sarcoid arthritis can reveal non-specific histological changes, including mild lining-cell proliferation, occasional vascular congestion, and diffuse infiltrates with lymphocytes and histiocytes, and may demonstrate granulomas. The effusion may be only mildly inflammatory, with distinctly less inflammation than, for example, in rheumatoid arthritis [36]. Synovial fluid examination can also be helpful in excluding other diagnoses, including infection such as tuberculosis.

Chronic sarcoid arthritis has a poorer prognosis than acute Lofgren’s; however, no consensus on a treatment approach exists [37]. Successful treatment with glucocorticoids, conventional synthetic disease-modifying antirheumatic drugs (csDMARDs), and biologic disease-modifying antirheumatic drugs (bDMARDs) has been reported.

### 4.3. Dactylitis

Dactylitis describes whole-digit swelling and the erythema of a finger or toe causing a sausage-like appearance, with associated pain and stiffness. In sarcoidosis, the underlying pathology can include tenosynovitis and enthesitis, often with granulomatous bone disease of the underlying phalanges. Subcutaneous sarcoidosis involving the digits and presenting as a dactylitis has also been reported [38]. Dactylitis occurs in fewer than 1% of cases of sarcoidosis, and is usually always associated with more widespread systemic involvement, often with skin disease such as lupus pernio [39]. Nail changes may occur when the distal phalanx is involved. Dactylitis is more common in those of African descent. Radiographs may demonstrate destructive bone changes with lytic lesions, trabecular lattice pattern, and cystic changes [40,41]. Management occurs within in the context of wider systemic sarcoidosis treatment, and sarcoid dactylitis usually responds well to glucocorticoids [41].

### 4.4. Jaccoud’s Arthropathy

Jaccoud’s arthropathy is a deforming, non-erosive type of arthritis, most commonly seen in autoimmune connective tissue diseases such as systemic lupus erythematosus. Cases of Jaccoud’s arthropathy have been reported in the context of systemic sarcoidosis and may occur later in the disease course rather than at initial presentation [42,43].

### 4.5. Osseous Sarcoidosis

Bone involvement in sarcoidosis has been reported in 1–15% of patients [31,44,45]. However, up to 50% of patients may be asymptomatic, therefore affecting reporting rates. A study on two large cohorts of patients with osseous involvement in sarcoidosis found a slight predominance in Caucasian patients and that osseous sarcoidosis was usually associated with more widespread systemic disease, with a high rate of pulmonary and extra-pulmonary involvement, including liver, spleen, and extra thoracic lymph node disease, as well as lupus pernio and uveitis [46]. The most commonly affected sites are the lumbar spine and pelvis, with a high prevalence of humerus, sacral, and femoral involvement, too. Involvement of the small bones of the hand is also frequently reported [20,31,45,47].

More sensitive imaging techniques, such as MRI or nuclear imaging, reveal a higher rate of bony involvement than plain radiography. MRI and ^18F^FDG-PET/CT demonstrate positive bone uptake in the early stages of osseous sarcoidosis [16]. Plain radiographic findings include osteosclerosis, lytic and cystic changes, or a trabecular latticework or “moth-eaten” appearance, particularly in the phalanges. Lytic lesions in more advanced osseous sarcoid usually involve the cortex and medulla, often of the middle and proximal phalanges [48]. Overlying soft tissue swelling and extensor tendon rupture may also occur. Where the distal phalanges are involved, associated overlying skin and nail disease may be seen [32]. Sclerotic lesions often affect the vertebrae—an important differential diagnosis is metastatic disease. In the absence of other typical features of sarcoidosis, biopsy is required to confirm the presence of non-caseating granuloma and to exclude malignant disease.

Low bone mass is associated with osseous sarcoidosis, with an increased risk of fragility fractures noted in the wrist, but not other sites [49]. Contributing factors to low bone mass include widespread skeletal granulomatosis, a high burden of glucocorticoid use, and the overproduction of calcitriol by the granulomas themselves, which can reduce bone mineralisation [50]. Despite bony disease, serum calcium and alkaline phosphatase levels are often normal [47]. Hypercalcaemia and hypercalciuria are seen in about half of patients, due to increased calcitriol production. The treatment of symptomatic cases involves systemic glucocorticoids, antimalarials, and surgery [51,52]. Successful treatment with tumour necrosis factor (TNF) inhibitors has also been reported [46]. As a result of the disease process and activation of osteoclasts, bone density can decrease, increasing fracture risk particularly in the distal forearm, a finding which is independent from but compounded by glucocorticoid use [49]. Vitamin D supplementation may be dangerous for some sarcoidosis patients and is recommended only for those with decreased 25-hydroxyvitamin D *and* a reduced or normal calcitriol level. The diagnosis and treatment of osteoporosis, and maintenance of bone health, are complex issues in sarcoidosis patients [14].

### 4.6. Axial Disease

A link between sarcoidosis and coexistent axial spondyloarthritis (AxSpA) has been reported, with two large studies reporting a prevalence of 6.6–14.3% in their sarcoidosis cohorts, compared to 1.9% in the general population [53,54]. Chronic back pain is a common symptom in sarcoidosis and there is a higher prevalence of AxSpA in patients with sarcoidosis and inflammatory back pain. Unilateral sacroiliitis may raise suspicion of granulomatous bone involvement, distinct from sacroiliitis. Spine and sacroiliac joint MRI is useful to investigate patients with sarcoidosis who report inflammatory back pain [55,56]. Asymptomatic vertebral involvement may also be discovered on imaging to assess other organ involvement in sarcoidosis, including ^18F^FDG-PET/CT. Most patients with vertebral involvement have a known diagnosis of intrathoracic sarcoidosis. Symptoms, if present, include mechanical pain or tenderness over the area. Vertebral lesions can appear lytic or sclerotic and in the absence of a known diagnosis of sarcoidosis can be difficult to distinguish from metastatic disease based on imaging alone; histological confirmation is therefore usually advised [57]. Vertebral bony lesions usually respond well to glucocorticoids.

### 4.7. Sarcoid Myopathy

It is estimated that muscle involvement occurs in up to half of patients with sarcoidosis, but the majority of cases are asymptomatic [58]. Non-caseating granulomas have been identified and reported in 50 to 80% of muscle biopsies of patients with active sarcoidosis, without clinical symptoms of myopathy [59]. Symptomatic muscle involvement usually occurs years into the disease course and affects up to 3% patients, usually in the context of other systemic features [60,61]. Various clinical subtypes of sarcoidosis-related myopathy are reported, including chronic myopathy, nodular myopathy, and acute myopathy syndromes.

#### 4.7.1. Chronic Myopathic Form

Chronic myopathy is the most frequently reported form, seen more commonly in females between the ages of 50 to 60 years. It presents with slowly progressive symmetrical proximal muscle weakness and atrophy, and the muscles of the trunk and neck may also be involved. The elevation of creatinine kinase only occurs in a few cases [62]. Imaging may show homogenous hyperintensity on T2-weighted MRI images with evidence of muscle atrophy. Electromyography (EMG) demonstrates a myopathic pattern. Granulomatous change is seen on muscle biopsy. Studies have shown that functional improvement after immunomodulatory treatment was significantly better in patients with short disease durations and the therapeutic response was poorer in those with a longer time to treatment, highlighting the importance of early diagnosis and the prompt initiation of treatment [62].

#### 4.7.2. Acute Myopathic Form

The rarest form of sarcoid myopathy is the acute myopathic type, which presents with proximal weakness and myalgia, sometimes with muscle swelling and pain [63]. It usually affects patients of a younger age, below 40 years, and tends to occur earlier on in the disease course [64]. Muscle enzymes are often elevated [61].

#### 4.7.3. Nodular Form

The nodular subtype presents with painful muscle nodules, with no motor deficit, and myopathic changes can be seen on EMG testing. MRI demonstrates intramuscular hypoechoic well-defined nodules, which are iso- or hyperintense relative to muscle on T1-weighted sequences. On T2-weighted images and Short Tau Inversion Recovery (STIR) sequences, lesions appear as intramuscular nodules of homogeneous high signal intensity. Nodules commonly disappear by the time of follow-up imaging after treatment with steroids [65].

#### 4.7.4. Other Sarcoid Myopathy Subtypes

Cohen Aubert et al. reported a large cohort of sarcoid myopathy and identified four alternative subtypes based on imaging and EMG findings: nodular, smouldering, myopathic, and a combined myopathic and neurogenic pattern [61]. Smouldering refers to myalgia without nodules or muscle atrophy on MRI, which is usually normal, but with evidence of myopathy on EMG testing; additionally, a moderate elevation in muscle enzymes is sometimes seen. The description of a combined myopathic and neurogenic type was based on the EMG findings, and muscle biopsy showed evidence of granulomas. Additionally, focal muscle involvement in sarcoidosis has been demonstrated [66].

A link to inclusion body myositis (IBM) has also been reported, with the frequency of IBM being higher in sarcoidosis than is seen in the general population [67]. IBM is less likely to respond to immunosuppressive treatment. Ensuring that alternative pathologies are excluded in patients presenting with myopathy in the context of sarcoidosis can help guide appropriate treatment [68].

#### 4.7.5. Investigation of Sarcoid Myopathy

In patients who present with both acute sarcoidosis and erythema nodosum, muscle biopsy will frequently show histological evidence of the disease [58]. The histopathological findings in sarcoid myopathy include non-caseating granulomas, muscle fibre changes which can be diffuse or anatomically related to granulomas, and histiocyte-associated damage of perimysial connective tissue [69]. In cases of sarcoid myopathy, there is usually clear evidence of systemic sarcoidosis elsewhere, but the differential diagnosis of unexplained granulomatous myopathy found on skeletal muscle biopsy includes inclusion body myositis, myasthenia gravis, autoimmune disorders including antineutrophilic cytoplasmic antibody (ANCA)-associated vasculitis, and T cell lymphoma [70]. Steroid-associated myopathy should also be considered in those patients presenting with muscle weakness after treatment with prolonged courses of glucocorticoids for sarcoidosis.

MRI is useful in detecting muscular sarcoid, evaluating the extent and distribution of muscle involvement, and monitoring the patient following glucocorticoid therapy [65]. Specific MRI findings include a star-shaped central structure of decreased signal intensity, a “dark star” sign, seen on axial images. A long nodule with an inner stripe of decreased signal intensity and outer stripes of increased signal intensity, a “three stripes” sign, may be seen on coronal and sagittal images [71]. A “tiger man” appearance of muscle oedema on either muscle MRI or ^18^FDG-PET/CT or nodular muscle abnormalities are both highly suggestive of muscular sarcoidosis [35].

## 5. Treatment

The management of systemic sarcoidosis presents a major clinical challenge due to the wide spectrum of disease manifestations. The decision of when to treat is based on the risk of organ failure and death in an individual patient, and the extent to which symptoms are impairing their quality of life. In most patients with sarcoidosis, the natural disease course is non-progressive and asymptomatic and may even resolve spontaneously without the need for specific treatment [3]. For example, in Lofgren’s syndrome, symptomatic treatment with non-steroidal anti-inflammatory drugs (NSAIDs) may be all that is required. In those with severe disease, however, timely treatment can reduce granulomatous inflammation and prevent long-term complications [72].

The lack of approved drugs tested in randomised controlled trials has precluded the development of standardised treatment protocols for sarcoidosis. Consensus statements endorse oral glucocorticoid treatment as the first-line treatment in systemic sarcoidosis [73,74]. Most of the evidence for the treatment of MSK manifestations is extrapolated from trials for lung disease. For joint disease, oral prednisolone 10–20 mg/day usually controls acute arthritis, and lower doses can be used for longer-term control of chronic disease [75]. However, there are dose-dependent adverse side effects which accumulate with long-term glucocorticoid use; therefore, the use of steroid-sparing agents is warranted for patients with persistent long-term inflammatory disease to prevent organ damage and/or treat symptoms. 

Common second-line steroid-sparing agents include methotrexate (MTX, used most commonly), azathioprine, leflunomide, and mycophenolate. Hydroxychloroquine, an antimalarial used commonly for other rheumatic diseases, has proved useful for cutaneous disease, acute arthritis, hypercalcemia, and some cases of neurosarcoidosis [26]. In sarcoid arthritis, MTX is likely to benefit those with evident synovitis rather than patients with tenosynovitis and periarticular swelling alone [23].

In the treatment of sarcoid myopathy, glucocorticoids remain the mainstay of treatment, as with other inflammatory myopathies, but a high rate of relapse is reported, particularly in the chronic forms [59]. MTX has been used as a steroid-sparing agent with some success for myopathy [76]. There have also been reports of the use of azathioprine and infliximab in the treatment of sarcoid myopathy. A large systematic review of myopathy in sarcoidosis reported that a high proportion of patients—around 50% at last follow-up—had permanent muscle weakness and replacement of muscle tissue by fat on MRI, thus highlighting a window of opportunity for early recognition and intensive treatment in muscular sarcoidosis, with the aim of preventing longer-term disability [66].

The majority of bone lesions in sarcoidosis are asymptomatic so do not require specific treatment, but osseous involvement often occurs in the context of multisystem sarcoidosis, which necessitates treatment. Glucocorticoids can improve the soft tissue swelling but do not change the underlying altered bone architecture [75].

If second-line csDMARDs are not tolerated or ineffective, then third-line treatment with biological DMARDs may be considered. Infliximab and adalimumab are both noted to be effective in the treatment of sarcoidosis. More recently, evidence from a small open-label trial has suggested that treatment with janus kinase inhibitor tofacitinib can improve sarcoidosis symptoms, predominantly by inhibiting type 1 immunity [77]. There has also been a case of successful treatment of steroid-resistant sarcoidosis with baricitinib [78]. A large study examining the prescribing practices in sarcoidosis in the US found that 12.1% of patients were treated with biologic or csDMARDs, and that biologic use remains highly variable across practices [79].

There does, however, appear to be a link between the use of biologic agents and the onset of sarcoidosis, with multiple case reports of TNF- and anti-interleukin 6-induced sarcoidosis when used in the treatment of other conditions. Clinicians must remain aware of the potential occurrence or reactivation of sarcoidosis when starting biologic treatment in patients with inflammatory arthritis. Further evaluation on larger cohorts is needed to investigate any causal link with the development of sarcoidosis [80,81,82].

## 6. Future Research Outlook

Treatment recommendations for the MSK manifestations of sarcoidosis are frequently extrapolated from the evidence base for treatment of pulmonary sarcoid or the management of other inflammatory arthritides. There is a paucity of randomised controlled trial (RCT) evidence for the management of rarer manifestations such as sarcoid myopathy. Further RCTs are also needed regarding the efficacy of bDMARD in treating MSK sarcoidosis, but the rarity of these disease manifestations poses a challenge for trial recruitment, and the clinical heterogeneity of sarcoidosis makes RCT design more difficult.

Currently, no validated disease activity tool or patient outcome measure exists for assessing treatment response across disease domains in sarcoidosis. Systemic sarcoidosis is a multifaceted disease which can be challenging to comprehensively assess, in particular those symptoms that are more subjective, such as fatigue. Recent patient surveys have emphasized that quality of life and function should be prioritized as outcome measures in treatment and care, and highlight the important role of multidisciplinary working in the management of sarcoidosis (Baughman et al., 2018 [83]). There have been recent RCTs evaluating potential treatments for fatigue in systemic sarcoidosis, which is a significant symptom and priority for patients (Atkins et al., 2021; Grongstad et al., 2020; Kahlmann et al., 2023 [84,85,86]).

## 7. Conclusions

Sarcoidosis is a multisystemic disease encompassing a wide spectrum of clinical presentations. The pattern of organ involvement varies, as does the clinical severity from a benign, self-limiting disease course with minimal symptoms to severe organ-threatening disease. Multiple different musculoskeletal manifestations can be seen in sarcoidosis including bone disease, acute arthritis, chronic arthritis, axial disease, and sarcoid inflammatory myopathy. They can occur as part of the initial presentation or appear later in the disease course, but usually within the first few years. Acute sarcoid arthritis has a very good prognosis and is normally a self-limiting condition, requiring symptomatic treatment only. Chronic arthritis and bone and muscle involvement usually occur in the context of more widespread multisystem disease. Asymptomatic muscle and bone involvement is common.

The diagnosis of sarcoidosis is based on a compatible presentation, combined with evidence of non-caseating granulomas on histology and the exclusion of alternative diagnoses, particularly infection, including tuberculosis, and malignancies, such as lymphoma. Rheumatologists are very well placed to assess a multisystemic disease which often presents a diagnostic challenge and providing oversight and follow-up of long-term immunosuppressive treatment. Treatment recommendations for the MSK manifestations of sarcoidosis are based on clinical experience and extrapolation from the evidence base for pulmonary sarcoidosis. There is a paucity of randomised controlled trial evidence for different treatments in MSK sarcoidosis. Glucocorticoids are beneficial in the treatment of joint and muscle disease in sarcoidosis, but second-line steroid-sparing treatment with conventional synthetic and biologic DMARDs may be required.

## Data Availability

No new data were created or analyzed in this study. Data sharing is not applicable to this article.
